# Historical Lassa Fever Reports and 30-year Clinical Update

**DOI:** 10.3201/eid1205.050052

**Published:** 2006-05

**Authors:** Abe M. Macher, Martin S. Wolfe

**Affiliations:** * US Public Health Service (retired), Bethesda, Maryland, USA;; † Travelers Medical Service of Washington, Washington, DC, USA;; ‡ George Washington University School of Medicine, Washington, DC, USA;; § Georgetown University School of Medicine, Washington, DC, USA

**Keywords:** Lassa fever, West Africa, Imported, Auditory, Deafness, Vestibular, Vertigo

## Abstract

Five cases of Lassa fever have been imported from West Africa to the United States since 1969. We report symptoms of the patient with the second imported case and the symptoms and long-term follow-up on the patient with the third case. Vertigo in this patient has persisted for 30 years.

Lassa fever is a viral hemorrhagic fever caused by a rodentborne arenavirus that is endemic in West Africa. In 2004, the Centers for Disease Control and Prevention (CDC) reported a fatal case of Lassa fever in New Jersey (*1*). CDC noted that ≈20 imported cases of Lassa fever had been seen outside West Africa. Five patients with imported disease have been hospitalized in the United States (Table). We report the signs and symptoms of the second patient in this group of 5 patients and the signs and symptoms and long-term follow-up of the third patient, both aid workers who became ill in 1975 while serving in Sierra Leone. Their clinical courses were complicated by severe neurologic dysfunction, including unilateral sensorineural deafness and vertigo.

**Table:** Patients with imported Lassa fever who were hospitalized in the United States*

Patient no.	Year of import	From	To	Clinical manifestations
1	1969	Nigeria	New York, NY	Fever, malaise, headache, nausea, sore throat, epigastric/right upper quadrant tenderness, pleural effusion, facial/cervical edema, dysphagia, elevated transaminases, cough, dyspnea, pulmonary infiltrates, epiglottal edema, lethargy, nystagmus, lightheadedness, dizziness without vertigo, ataxia, alopecia (*2*)
2	1975	Sierra Leone	Washington, DC	Abdominal pain, diarrhea, fever, headache, myalgia, arthralgia, conjunctival injection, lymphadenopathy, weight loss, pleuritic chest pain, pleural effusion, unilateral deafness
3	1976	Sierra Leone	Washington, DC	Abdominal cramps, nausea, vomiting, diarrhea, fatigue, headache, retroorbital pain, neck/back pain, paresthesias, right ear pain, fever, vertigo, syncope, dysmorphopsias, alopecia, weight loss, ecchymoses, insomnia, depression, hypotension, left-sided facial weakness, right-sided Babinski reflex, Weber test lateralized to the left (*3*)
4	1989	Nigeria	Chicago, IL	Shaking chills, fever, sore throat, myalgia, headache, dysphagia, bloody diarrhea, elevated transaminases, hypotension, adult respiratory distress syndrome, death (*4*)
5	2004	Sierra Leone and Liberia	Trenton, NJ	Chills, fever, sore throat, diarrhea, back pain, adult respiratory distress syndrome, death (*1*)

*Patients 1–4 are US citizens; patient 5 is a Liberian national.

## Case 1

In February 1975, a 26-year-old American aid worker in Sierra Leone was hospitalized with severe abdominal pain. No cause was determined, and she was discharged. In March 1975, watery diarrhea, fever, chills, headache, myalgias, arthralgias, and conjunctival injection developed. She was hospitalized, and physical examination showed posterior cervical, axillary, and inguinal lymphadenopathy. She was empirically treated for malaria and amebiasis.

Nevertheless, fever persisted, she lost 2.7 kg, and pleuritic chest pain developed. In April 1975, she was air evacuated and admitted to a hospital in Washington, DC. Although she was afebrile, generalized lymphadenopathy was still present, and a chest radiograph showed left-sided pleural effusion. Thoracentesis fluid was remarkable for eosinophilia, and examinations of blood showed 3%–35% peripheral eosinophilia. Knott's preparation of blood showed 3 sheathed microfilariae with nuclei extending into the tail, presumed to be *Loa loa*. A cervical lymph node biopsy showed follicular hyperplasia.

She was convalescing in the hospital when suddenly, while speaking on the telephone, she lost hearing unilaterally. An audiogram demonstrated unilateral sensorineural deafness. A serum specimen collected in May 1975 was sent to CDC, where an indirect fluorescent antibody (IFA) titer of 256 was demonstrated against Lassa fever virus (P. Rollin, pers. comm.). She was discharged with residual unilateral deafness.

## Case 2

In December 1975, abdominal cramps, nausea, vomiting, diarrhea, fatigue, headaches, retroorbital pain, aching shoulders, and severe low back pain developed in a 43-year-old American aid worker in Sierra Leone.^1^ Her aching progressed to total body pain, which she described as "severe pain in her bones, as if they were breaking" (from patient's medical chart). Her symptoms persisted, and in February 1976, nocturnal fevers and sweats developed. She experienced dizziness and syncope and was hospitalized. She was hypotensive with blood pressure as low as 70/40 mm Hg (compared to 120/80 mm Hg in June 1975) and had insomnia. She was empirically treated for malaria and discharged. Her symptoms reappeared, accompanied by persistent vomiting, shooting pain in the right ear, neck pain, paresthesias, and alopecia. She lost 4 kg. In March 1976, she was air evacuated and admitted to a hospital in Washington, DC.

During her hospitalization in Washington, she was afebrile. However, fatigue, headache, neck pain, nausea, low back pain, and insomnia persisted. She had costochondral and diffuse abdominal tenderness and ecchymoses at intramuscular injection sites (antiemetics). She was unable to read for more than a few minutes, as her eyes would tire and begin to hurt. She experienced dysmorphopsias, difficulty with hearing, severe depression, and numerous episodes of lightheadedness, unsteadiness, dizziness, and vertigo. Vertigo occurred in both supine and standing positions up to 5 times per day. Although she was hypotensive, she was not orthostatic. Neurologic examination found left-sided facial weakness, right-sided Babinski reflex, and the Weber test lateralized to the left. Audiometry and positional and caloric nystagmography results were unremarkable.

A serum specimen obtained on March 1 showed an IFA titer of 64 against Lassa virus. Lassa virus was recovered from a March 3 urine specimen. On March 10, a serum specimen demonstrated a complement fixation antibody titer of 16, a 4-fold rise compared to a titer <4 in a February 25 specimen drawn in Sierra Leone.

Although her vertigo persisted, she became normotensive (120/80 mm Hg) on March 28, 1976, and was discharged. However, during the next 30 years, she continued to experience fatigue, generalized weakness, headache, insomnia, depression, dysmorphopsias, paresthesias, lightheadedness, dizziness and syncope, and labile hypotension. She describes "fatigue so severe that I have no energy for days," "staggering when getting up," "inability to produce words at times," and "spells of loss of consciousness" (up to 15 minutes in duration, as noted by her husband). In 1992, a magnetic resonance imaging scan of the brain demonstrated periventricular hyperintense signals. As of February 2006, her symptoms persist.

## Conclusions

Auditory or vestibular dysfunction may develop in patients with Lassa fever, and tinnitus, autophony, hearing loss, dizziness, vertigo, nystagmus, and ataxia have been reported (*3**,**4*). In their review of a 1989 nosocomial Lassa fever outbreak in a Nigerian hospital, Fisher-Hoch et al. (*3*) noted a high fever in the index patient, who was taken to surgery on February 25. The patient bled profusely and died later that night. The surgical nurse and a student nurse who washed blood-soaked cloths both became ill with febrile illnesses on March 7. Both became serologically positive for Lassa fever virus. The surgical nurse was traced to her village, where she was found to be almost totally deaf and severely ataxic.

Onset of deafness among patients with Lassa fever is a feature of the convalescent phase rather than the acute phase of the illness (*4*). Deafness was first reported as a complication of Lassa fever by White (*5*) and Henderson (*6*) in 1972. White noted that during a 1970 nosocomial hospital outbreak in Jos, Nigeria, deafness occurred in 4 of 23 hospitalized patients; a fifth patient reported intermittent tinnitus, and 3 patients experienced dizziness.

Among the now 24 reported patients with imported Lassa fever worldwide (1969–2004, Table A1, our 26-year-old aid worker is the only patient whose clinical course has been complicated by sensorineural deafness. Our second patient's clinical course has been remarkable for an array of acute and chronic neurologic and neuropsychiatric complications, including left-sided facial weakness, right-sided Babinski reflex, headache, paresthesias, vertigo, syncope, dysmorphopsias, fatigue, insomnia, and depression. Rose (*7**,**8*) reported a 1955–1956 outbreak of encephalomyelitis in Sierra Leone, which may represent the earliest recorded clinical description of Lassa fever; remarkably, vertigo developed in 30 of his 45 patients. Solbrig and McCormick (*9*) reported that neuropsychiatric sequelae of Lassa fever have included sleep disorders (e.g., insomnia), asthenia, multiple somatic complaints, psychosis, hallucinations, personality disorders, severe adjustment reactions, dementia, mania, and depression. Finally, our patient's ongoing labile hypotension may represent Lassa fever–induced damage to the brain stem with resultant autonomic dysfunction. Since our patient's array of persistent neurologic and neuropsychiatric symptoms have not changed, improved, or progressed since her episode of Lassa fever, we believe that they all may represent sequelae of Lassa fever–induced damage to the brain.

## Footnotes

1 Portions of this patient's clinical signs and symptoms were originally published in Zweighaft et al. (*2*).

## Appendix

**Table A1:** Patients with imported Lassa fever, worldwide, 1969–2004

Year of import	From	To	Occupation	Clinical outcome	Reference
1969	Nigeria	United States	Nurse	Survived	(*10*)
1971	Sierra Leone	United Kingdom	Nurse	Survived	(*11*)
1971	Sierra Leone	United Kingdom	Physician	Survived	(*11*)
1972	Sierra Leone	United Kingdom	Nurse	Survived	(*12*)
1974	Nigeria	Germany	Physician	Survived	(*13*)
1975	Nigeria	United Kingdom	Physician	Died	(*13*)
1975	Sierra Leone	United States	Aid worker	Survived	
1976	Sierra Leone	United States	Aid worker	Survived	(*14*)
1976	Nigeria	United Kingdom	Engineer	Survived	(*15*)
1980	Upper Volta	Netherlands	Aid worker	Survived	(*16*)
1981	Nigeria	United Kingdom	Teacher	Survived	(*17*)
1982	Nigeria	United Kingdom	Diplomat	Survived	(*18*)
1984	Sierra Leone	United Kingdom	Geologist	Survived	(*19*)
1985	Sierra Leone	United Kingdom	Nurse	Survived	(*20*)
1987	Sierra Leone/Liberia	Israel	Engineer	Survived	(*21*)
1987	Sierra Leone	Japan	Engineer	Survived	(*22*)
1989	Nigeria	Canada	Agricultural specialist	Survived	(*23*)
1989	Nigeria	United States	Engineer	Died	(*24*)
2000	Cotê d'Ivoire/Burkina Faso/Ghana	Germany	Student	Died	(*25*)
2000	Sierra Leone	United Kingdom	Peacekeeper	Died	(*25*)
2000	Nigeria	Germany	Unknown	Died	(*25*)
2000	Sierra Leone	Netherlands	Physician	Died	(*25*)
2003	Sierra Leone	United Kingdom	Peacekeeper	Survived	(*26*)
2004	Sierra Leone/Liberia	United States	Businessman	Died	(*27*)
